# Variants of Callous–Unemotional Traits in Middle Childhood: An Investigation of Emotional Regulation, Externalizing Behaviors, and Psychosocial Risk Factors

**DOI:** 10.3390/children12070835

**Published:** 2025-06-25

**Authors:** Yu Gao, Ines Guariguata, Liat Kofler

**Affiliations:** 1 Brooklyn College, City University of New York, New York, NY 10065, USA; 2 Graduate Center, City University of New York, New York, NY 10065, USA; iguariguata@gradcenter.cuny.edu (I.G.); lkofler@gradcenter.cuny.edu (L.K.)

**Keywords:** psychopathy, callous, antisocial, emotion processing, autonomic nervous system, youth

## Abstract

**Background**: A growing body of literature suggests that there are two variants of callous–unemotional (CU) traits—primary (with low anxiety) and secondary (with high anxiety)—although whether these traits differ in emotional regulation is unknown. The present study aimed to further our understanding of these variants by comparing the two variant groups (high CU/low anxiety and high CU/high anxiety) with two control groups (low CU/low anxiety and low CU/high anxiety) on emotional regulation, along with a variety of psychosocial and externalizing behavioral measures. **Methods**: A community sample of children aged 8 to 10 years (*N* = 340) from the northeast United States and their main caregivers participated in this study. Children completed an emotional regulation task while their sympathetic and parasympathetic nervous system activities were recorded. Prenatal and postnatal adversity and externalizing behaviors of the children were assessed using caregiver and child reports. **Results**: It was found that children with high CU/high anxiety were characterized by elevated parasympathetic activities during the emotional regulation task, along with more adversity and externalizing behaviors. **Conclusions**: These findings extend our knowledge on CU by highlighting the emotional dysregulation and more severe clinical picture that were associated with more psychosocial adversity in the high CU/high anxiety variants.

## 1. Introduction

As a developmental precursor to adult psychopathy, callous–unemotional (CU) traits in youth refers to a cluster of characteristics including lack of remorse or empathy, callous use of others, and shallow or deficient affect [1,2]. Youths with higher levels of CU traits comprise a subgroup characterized by more severe and aggressive behavioral problems than those with fewer CU traits [1]. A growing body of literature suggests that there are two variants of CU traits: the primary variant, characterized by low anxiety, reduced sensitivity to distress, and early temperamental and biological dysfunctions; and the secondary variant, which is characterized by high anxiety and theorized to be predominantly influenced by environmental factors [3]. Evidence suggests that these two variants are distinguishable as early as 3 years old [4], and that these traits are relatively stable in childhood [5,6]. However, in one recent study, researchers were able to distinguish the two variants in school-aged but not in preschool children [7]. In the current study, we examined a wide variety of psychosocial and psychophysiological factors in a community sample of boys and girls who showed various levels of CU traits. We focused on the emotional regulation deficits associated with the two variants. Given that the distinction between CU variants is rooted in the adult psychopathy literature, we aimed to enhance the understanding of these variants in middle childhood by identifying the common and distinct risk factors associated with each variant, which would inform the etiology and improve the treatment of psychopathic and antisocial behaviors.

### 1.1. Emotional Regulation Deficits and CU Traits

Emotional regulation refers to the processes by which individuals respond to and modulate (e.g., amplify, maintain, or decrease) their emotional responses in order to adapt to emotional contexts [8]. In particular, it refers to all processes involved in shaping which and when emotions are experienced and expressed [9], whereas any pattern of emotional experience or expression that interferes with goal-directed activity is defined as emotional dysregulation [10]. Emotional regulation is a learned skill acquired through early parent–child interactions, and it relies on the intentional strategies a person uses after the initial experience of an emotional response [11,12]. Despite its critical importance to youth development, research exploring emotional regulation in children with CU traits has been limited. One study in a nonclinical sample of children aged 6–11 years (*N* = 194) found that caregivers of children with high callousness and uncaring scores reported poorer emotional regulation skills in their offspring [13]. Furthermore, children with poorer emotional regulation were more likely to find immoral acts acceptable, suggesting that deficits in emotional regulation may partly contribute to the elevated antisocial behavior observed in children with high CU traits [14]. In another study, researchers utilized psychophysiological measures to examine emotional regulation capacity in children with attention deficit hyperactivity disorder (ADHD) and low prosocial behavior, which was used as a proxy for high CU traits [15]. In the study, children aged 7–11 years were asked to facially mimic (induce) or hide (suppress) emotions experienced by characters in movie clips. They found that the ADHD + high CU group displayed blunted sympathetic and parasympathetic reactivity across task conditions, suggesting that low emotional arousal may reflect emotional dysregulation in children with high CU traits.

Very few studies have examined emotional regulation in the two variants of CU traits. In a clinical sample of 418 adolescents (ages 12–19 years), researchers conducted a latent profile analysis which revealed a four-profile model that differentiated CU variants based on emotional regulation differences: primary CU (high CU/low dysregulation), secondary CU (high CU/high dysregulation/maltreatment), anxious (low CU/high dysregulation), and low risk (low CU/low dysregulation; [12]). The primary variants were characterized by underarousal, including low anxiety and low affect dysregulation, whereas the secondary variants had higher levels of affect dysregulation, affect suppression, anxiety, and maltreatment. It is important to note that as the study examined CU variants in a clinical sample, findings may not generalize to non-clinical, non-incarcerated community populations. In addition, since emotional regulation was used to profile participants, the study does not inform as to whether the two traditionally defined variants (based on anxiety) differ in emotional regulation.

Evidence has suggested that when processing emotional stimuli, the high CU/low anxiety and high CU/high anxiety variants are characterized by hypo- and hyper-emotional reactivity, respectively. For example, one study found that children with high CU/high anxiety had higher heart rates, reflecting heightened emotionality [4]. In another study by the same research group, researchers used a multi-system physiological approach (i.e., startle potentiation, heart rate, skin conductance, and medial prefrontal activity) to investigate heterogeneity among children and young adults distinguished by CU traits, conduct problems, and anxiety [16]. Although the groups did not differ on their baseline heart rate or skin conductance activity, children with high CU/high anxiety showed a larger startle reactivity and physiological arousal to violent, fearful, and anger stimuli compared to those with high CU/low anxiety. Nonetheless, no study has examined emotional regulation, using psychophysiological or other measures, in the context of CU variants. Given its high comorbidity with psychopathology, particularly anxiety, individuals with high CU/high anxiety were expected to exhibit emotional dysregulation. In contrast, no emotional regulation deficits were expected to be found in those with high CU/low anxiety.

### 1.2. Psychosocial Risk Factors and CU Traits

Previous research has consistently demonstrated that individuals with high CU/high anxiety experience greater adversity than those with high CU/low anxiety. In a recent review, researchers reported that over 80% of the included studies found that, compared to individuals with high CU/low anxiety, those with high CU/high anxiety had higher rates of abuse/trauma or adverse childhood experiences, including parental absence, domestic violence, physical, emotional and sexual abuse, as well as family adversity, harsh parenting, and maternal psychopathology [17]. In addition, parents of children with high CU/high anxiety show higher rates of intimate partner violence and stress-related psychopathology, which are associated with an increased risk of engaging in hostile, rejecting, detached, and unresponsive parenting, as well as maltreatment [4,18,19,20]. However, one study failed to find differences in parental negativity or harsh discipline between the two variants [21].

Studies have also found that different types of abuse experienced during development may have unique associations with CU variants [17]. For example, one study found that incarcerated male adolescents with high CU/high anxiety had experienced higher rates of sexual but not emotional or physical abuse, while those with high CU/low anxiety had higher rates of emotional and physical neglect [22]. Another study found that individuals with high CU/high anxiety showed higher levels of emotional, physical, and sexual abuse, as well as physical neglect, compared to those with high CU/low anxiety [23]. To date, only one study has compared youths with the two CU variants to low-risk youth from the community, and the findings demonstrated that both variants showed a greater severity of maltreatment histories than low-risk youth, although the two variants did not differ from each other [24]. In the current study, we compared children with either CU variant to those with low CU traits on subtypes of maltreatment.

Relatively little is known regarding whether the two CU variants are differentially associated with prenatal risks, although prenatal maternal psychopathologies, including anxiety and depression, have been linked to increased CU at age 13 years [25]. One study found that individuals with high CU/high anxiety experienced the highest levels of family adversity (including both socioeconomic disadvantage and interpersonal stressors) and maternal psychopathology (anxiety, depression) starting in pregnancy [26]. Given that a large body of the literature finds that prenatal maternal stress is associated with atypical fetal development, which in turn increases offspring susceptibility for postnatal disease and maladjustment [27], it has been argued that the increased range of psychopathologies in those with high CU/high anxiety can be partially attributed to this prenatal exposure [26]. It was therefore hypothesized in the current study that children with high CU/high anxiety would show more prenatal risks than those with high CU/low anxiety and those with low CU traits.

### 1.3. Comorbid Psychopathology

The extant literature highlights greater comorbid psychopathologies for individuals with high CU/high anxiety compared to their low-anxiety counterparts [28,29], including a greater frequency and severity of reactive externalizing behaviors, delinquency, and violent offenses across the lifespan [5,6]. In one study, researchers examined a sample of incarcerated adolescent male offenders and found that, compared to youths with high CU/low anxiety, youths with high CU/high anxiety engaged in significantly more violent behaviors during incarceration (69.4% vs. 92%) and had higher variability in their violent behavior across years [30]. In addition, their violent behavior was more likely to be reactive in nature (i.e., responding angrily to a perceived provocation: 82% of violent incidents vs. 54% in the low anxiety group). Similarly, another study found adolescents with high CU/high anxiety to have higher levels of reactive aggression, but similar levels of proactive aggression, compared to their low-anxiety counterparts [29]. In contrast, a study of high-risk females found that those with primary and secondary variants were not differentiated on their self-reported levels of proactive and reactive relational aggression [6]. In summary, while individuals characterized by either variant appear more likely to engage in aggressive and criminal behavior, some preliminary findings show that those with high CU/high anxiety may have higher rates of reactive aggression, which may be related to their elevated levels of impulsivity and/or cognitive and regulatory deficits.

### 1.4. Summary

In conclusion, while a growing body of research supports the differentiation of two distinct variants of CU traits based on levels of anxiety, with unique developmental trajectories and characteristics, the distinction between the two remains insufficiently addressed in the literature, particularly in school-age samples. Moreover, a significant proportion of previous studies have predominantly focused on male youths involved in the justice system [17], and more studies are needed to examine the distinction between variants in mixed-gender and community samples to enhance the generalizability of the findings. The current study aimed to address these gaps by comparing a wide variety of psychosocial and psychophysiological measures between the two variants and controls in a group of children from the community, with a focus on emotional regulation deficits. It was hypothesized that children with high CU/high anxiety would be characterized by emotional dysregulation, more post- and prenatal adversity, and more comorbid psychopathologies along with reactive aggression, as compared to children with high CU/low anxiety and children with low CU traits. Finally, prior studies have used anxiety, maltreatment or post-traumatic stress, or the combination of both anxiety and maltreatment, as distinguishing features to identify these two variants (see reviews by [17,24]). However, one recent study demonstrated that anxiety should be used as the distinguishing feature alongside CU traits when defining the primary and secondary variants, since models using anxiety have yielded better fitting and more theoretically interpretable classification than models using maltreatment [24]. Therefore, in the current study, anxiety was used to identify the two variants.

## 2. Materials and Methods

### 2.1. Participants

Participants in this study were recruited as part of the Healthy Childhood Study, a longitudinal study examining the development of behavioral problems from childhood through late adolescence. Participants consisted of 340 children from Brooklyn, New York (mean age = 9.06 years, SD = 0.60, 51.8% female), with no diagnosed psychiatric disorder, pervasive developmental disorder, or intellectual disability. The ethnic/racial breakdown is 52% African American, 21% Caucasian, 11% Hispanic, 2% Asian, and 14% mixed race/other. The caregivers of the participants were mostly biological mothers (86.4%), with 59% of the children living with both biological parents, and 29% living alone with their biological mother. More information about the sample can be found in [31].

### 2.2. Procedure

Participants and their caregivers were assessed during a 2 h laboratory visit at the university. During the visit, children and their primary caregivers underwent psychophysiological testing, neurocognitive and psychosocial assessments, and a behavioral interview. Caregivers gave consent and permission, and children gave verbal assent before participation. Then, children were administered several tasks while their psychophysiological responses were obtained. Behavioral interviews and neurocognitive and psychosocial assessments were administered by trained research assistants either before or after the psychophysiological testing session. An assistant was present with the child throughout the testing session while caregivers completed their questionnaires in a separate room. Each participating family received monetary compensation in addition to transportation reimbursement. All procedures were approved by the University Institutional Review Board.

Participants’ demographic information was assessed via caregivers’ reports. Children’s intelligence was assessed using the four subsets of the Wechsler Intelligence Scale for Children, 4th edition (WISC-IV; [32]). Participants completed four subtests (Vocabulary, Matrix Reasoning, Digit Span, and Coding) and scores were prorated and summed to estimate their full-scale IQ.

### 2.3. Emotional Regulation Task

The psychophysiological testing session lasted approximately 40 min and consisted of several tasks, including an emotional regulation task and two 2 min rest periods (one at the onset, the other at the end of testing) during which children were instructed to quietly relax and remain still. The emotional regulation task was modified based on the paradigm used in [33]. In this task, children watched four 2 min film clips from the movie Homeward Bound. They were instructed to imagine the emotion of the main characters, and to either facially mimic the emotion (induction) or keep their face still (suppression) during the clips. The first two clips were intended to elicit negative emotions, and the last two elicited positive emotions. The sequence of the four clips was as follows: (1) negative induction, (2) negative suppression, (3) positive induction, (4) positive suppression. The order of these clips was kept constant for each participant in order to end with positive emotions, and to keep the same order as prior studies [33]. A 2 min rest period between the second and the third clips was provided to restore a baseline. Psychophysiological data were recorded continuously during the task. Children reported their emotional experiences using a Self-Assessment Manikin (SAM; [34]) in terms of valence (how positive or negative the emotion was) and arousal (the intensity of the emotion) after each movie condition [33]. Higher scores indicate more positive emotions and a higher intensity of the emotion.

### 2.4. Measures

#### 2.4.1. Group Formation

- Callous–Unemotional Traits

Each child and caregiver completed the 24-item self-report and caregiver versions of the ICU questionnaire [35], respectively. Caregivers/children were asked how well specific statements described their child/themselves. All items were rated on a 4-point Likert scale from 0 (not true at all) to 3 (very true) and were summed to create total scores. The ICU consists of three subscales: callousness (11 items, e.g., does not seem to know “right” from “wrong”), uncaring (8 items, e.g., feeling bad or guilty when he/she has done something wrong), and unemotional (5 items, e.g., expresses his/her feelings openly). The higher score between the child’s report and the caregiver’s report was used as the final score for each child, as recommended by the ICU and Antisocial Process Screening Device Manual [36]. Cronbach’s alpha was 0.85 for the total CU score in the current sample [37].

- Anxiety

The level of anxiety was determined by relevant items in the Child Behavior Checklist (CBCL; [38]). The CBCL is a rating scale consisting of 112 items asking caregivers to report on their child’s behavior over the last 12 months. It uses a 3-point Likert scale: 0 = not true, 1 = sometimes true, 2 = very true/often true. Anxiety was assessed using the subscale based on the DSM-oriented anxiety items [39]. The DSM-based subscales in general population/non-referred samples have been assessed for validity in prior studies [40].

The groups were formed following procedures used in prior studies [41]. First, participants with high CU (top third) or low CU (bottom third) traits were identified using a tertile split on the CU score. Next, these participants were separated into high anxiety and low anxiety based on the median split of their anxiety score. Therefore, four groups were formed: control (low CU/low anxiety), anxious (low CU/high anxiety), primary CU (high CU/low anxiety), and secondary CU (high CU/high anxiety).

#### 2.4.2. Psychosocial Risk Factors

- Prenatal Maternal Stress

Maternal psychological distress during pregnancy was retroactively assessed via a questionnaire that included the following stressful event items: job changed/fired from job, natural disaster, being a crime victim, trouble with the law, illness or death of relatives, illness or death of significant other, divorce/separation, moving, financial problems, accident, conflicts with significant others, own physical illness, and own mental illness. One point was scored if each of the above had happened during the pregnancy. Caregivers also indicated “yes”, “no”, “not at all”, “somewhat”, or “very” to questions such as “How happy did the pregnancy make you (negatively coded)?”; “Were you depressed after birth?”; “Was this pregnancy planned (negatively coded)?”. These were then coded dichotomously as either present (“yes”, “somewhat”, “very”) or absent (“no”, “not at all”). Scores were summed to create a measure of prenatal maternal stress, with a range between 0 and 10, a median of 2, and an internal consistency of 0.78.

- Social Adversity

The social adversity index assesses the cumulative level of social adversity experienced by each family and was assessed based on 10 variables derived from prior research [42,43]. The 10 variables are as follows: divorced parents (single parent family, living with guardians other than parents, or remarriage), foster home, welfare food stamps, teenage mother (aged 19 or younger at the time of child’s birth), crowded home (five or more family members per room within the home), parent ever arrested (either parent was arrested at least one time), large family (sibling order fifth or higher by the time the child was 3 years old), parents mentally ill, parents physically ill, and public housing. Each of the 10 variables was scored as either “yes” (1) or “no” (0). A total score was computed by summing the number of “yes” responses, with a higher score representing greater social adversity.

- Exposure to Childhood Abuse and Domestic Violence

Exposure to physical and verbal childhood abuse was assessed using a modification of the Conflict Tactics Scale [44] completed by the caregiver. This modified measure has been validated with adults who had been physically abused 20 years prior as demonstrated by official court reports of child abuse, and shows good discriminant and predictive validity [45]. It is a 5-point Likert scale (0 = never, 1 = once, 2 = twice, 3 = sometimes, 4 = frequently, 5 = most of the time) about the parents’ behavior toward their child when disagreement occurs. Total scores of physical abuse (nine items including throw something at your child, push/grab/shove, slap/spank, kick/bite/hit with a fist, hit/try to hit your child with something, beat up, choke, threaten with knife/gun, use a knife/gun) and verbal abuse (six items including insult or swear, sulk and/or refuse to talk, stomp out, do or say something to spite your child, threaten to hit or throw something at your child, throw/smash/hit/kick something) were computed [46]. The coefficient alpha was 0.73 for the physical abuse measure and 0.71 for the verbal abuse measure. Similar questions were asked to the caregivers about their spouses’ behavior when disagreement occurred. Total scores of domestic violence (physical or verbal) were computed as listed above, with coefficient alphas ranging from 0.83 (verbal violence) to 0.93 (physical violence).

- Parent Criminal Behavior

Caregiver’s criminal offending history was assessed on a modified version of the Self-Report Crime Checklist (SRCC; [47]). Each caregiver answered 31 questions related to a range of criminal offences. White-collar criminality and other criminality scores were generated by summing the relevant items. Examples of white-collar crimes were as follows: used computers to illegally gain valuable information or gain money; conned a person or business for financial gain; obtained health or unemployment benefits through lying; stole work supplies; impersonated another person for personal gain. Other crimes included items such as stole things from markets; broke into a car; carried a weapon like a knife or used a weapon in a fight; threatened to kill someone. Items from the original SRCC were re-coded as follows to reflect the number of times an offence was committed: 0 (never), 1 (once), 2 (at least twice). These translated to the classifications of “never offended”, “one-time offender”, or “recidivistic offender”, and each item was summed to create a total aggregate score for the white-collar and other crimes, respectively [47].

- Neighborhood Collective Efficacy

Caregivers reported their neighbor’s trustworthiness and willingness to intervene for the sake of the neighborhood by answering 10 questions that assess social control and cohesion [48]. It is a 5-point Likert scale (“strongly disagree” to “strongly agree”), and example questions include “children were skipping school and hanging out on a street corner (reverse coded)” and “people around your neighborhood are willing to help neighbors”. All items were summed to produce a total score for overall collective efficacy, with a Cronbach’s alpha of 0.90 for the overall sample. More information can be seen in [49].

#### 2.4.3. Externalizing Behavior Measures

- ADHD/ODD/CD symptoms

These symptoms were assessed using the Diagnostic Interview Schedule for Children (DISC-IV; [50]), a computerized semi-structured interview that has been found reliable and valid in prior studies [51]. Caregivers were administered the DISC-IV to assess the child’s lifetime number of ADHD, CD, and ODD symptoms. None of the participants met the diagnostic criteria for CD or ODD; however, CD and ODD symptoms were present. For ODD symptoms, over 80% of children displayed one or more lifetime symptoms. In terms of CD symptoms, over 28% of children had one or more symptoms. Finally, over 31% of children had one or more ADHD symptoms.

- Reactive and Proactive Aggression

Children completed the Reactive-Proactive Aggression Questionnaire (RPQ; [52]), which consists of a total of 23 items, with 11 assessing reactive aggression (e.g., “yelled at others when they have annoyed you”) and 12 assessing proactive aggression (e.g., “had fights with others to show who was on top”). They rated the frequency of each occurrence on a scale of 0 to 2: 0 = never, 1 = sometimes, 2 = often. Each subscale was calculated by summing the scores across all items, with a higher score reflecting a higher level of aggression. In the current sample, the internal reliability was 0.82 and 0.81 for reactive and proactive aggression, respectively.

- Aggression and Delinquency

Aggressive and rule-breaking behaviors were assessed using the Child Behavior Checklist (CBCL; [38]) and Youth Self-Report (YSR; [53]). The CBCL is a 112-item rating scale that caregivers complete to rate different components of a child’s behavior within the last 12 months. Example items include “argues a lot” and “impulsive or acts without thinking”. Items are rated on a 3-point scale as follows: 0 = not true, 1 = sometimes or somewhat true, 2 = very true or often true. Subscale scores were the sums of raw scores for each item. Only the aggression and delinquency subscale scores were included in the analysis for the purposes of this study. In the current sample, Cronbach’s alpha was 0.87 and 0.71 for aggression and delinquency, respectively. Children completed the YSR [53], a scale of 118 items rated on a 3-point scale (0 = not true, 1= sometimes or somewhat true, and 2 = very true or often true), each describing problems specific to themselves. The coefficient alphas for the present sample were 0.79 and 0.49 for the aggression and delinquency subscales, respectively.

- Impulsivity and Narcissism

Both children and caregivers completed the impulsivity (5 items, e.g., “acts without thinking”) and narcissism (7 items, e.g., “thinks he or she is more important than others”) subscales of the Antisocial Process Screening Device (APSD; [36]), a scale developed to assess psychopathic traits in youth. The child and caregiver each responded on a three-point scale ranging from 0 (not at all true) to 2 (definitely true). Scores in each subscale were summed to form the corresponding measures. The reliability and construct validity of APSD have been supported in numerous samples (e.g., [54]). In the current sample, the reliability was acceptable for both the child’s (0.72 for impulsivity and 0.74 for narcissism) and caregiver’s report (0.76 for impulsivity and 0.73 for narcissism).

### 2.5. Psychophysiological Data Acquisition and Reduction

A BIOPAC MP150 system (BIOPAC systems Inc., Goleta, CA, USA) was used to collect all psychophysiological data, and data were analyzed offline using AcqKnowledge 4.2 software. At the onset and at the end of the computerized tasks, participants sat for 2 min rest periods, when children were instructed to sit quietly. If participants’ data was unavailable or unsuitable because of movement, only the available rest period data was used for analysis.

#### 2.5.1. Respiratory Sinus Arrhythmia (RSA)

RSA data was derived from an ECG100C amplifier at 1000 Hz with a band pass filter of 35 Hz and 1.0 Hz, as well as an RSP100C respiration amplifier with a band pass filter of 1.0 Hz and 0.05 Hz. Two pre-gelled Ag-AgCl disposable vinyl electrodes were placed in a modified Lead II configuration on each participant. The electrocardiogram (ECG) signals were visually inspected for artifacts. The automated AcqKnowledge 4.2 modified Pan-Tompkins QRS detector converted the ECG signal to R-R intervals, then the AcqKnowledge automated RSA analysis was applied. Analysis followed the well-validated peak–valley method [55], in which RSA is calculated (ms) as the difference between the minimum and maximum R-R intervals during respiration.

#### 2.5.2. Pre-Ejection Period (PEP)

Data was derived offline from the ECG and impedance cardiography (ICG) signals recorded using a NICO100C amplifier. Eight spot electrodes were placed on participants in the following configuration: two on each side of the upper neck and two on each side of the lower torso, in accordance with previous research [56]. Recorded ECG and ICG signals were inspected on a beat-to-beat basis and ensemble-averaged. The automated scoring algorithm measured PEP (ms) from the onset of the ECG Q-wave to the B-point of the dZ/dt wave.

Resting psychophysiological measures were calculated as the average of the 2 min resting periods at the beginning and end of the testing sessions. All values for both resting periods were highly correlated, thereby supporting the calculation of an average baseline score. For each psychophysiological index, reactivity measures were calculated by subtracting baseline measures during the rest from task measures during each movie condition. For RSA reactivity, higher values reflect increased PNS activity during the task (PNS activation), while lower values indicate a reduction in PNS activity (PNS inhibition). For PEP reactivity measures, negative scores indicate a shortened PEP and increased SNS reactivity (SNS activation), while positive scores denote less PEP shortening or reduced SNS reactivity (SNS inhibition).

### 2.6. Statistical Analyses

All analyses were conducted using SPSS 29 (SPSS Inc., Chicago, IL, USA). To test for group differences in emotional regulation, a repeated-measures analysis of variance (ANOVA) was conducted with the group (control (low CU/low anxiety), anxious (low CU/high anxiety), primary CU (high CU/low anxiety), and secondary CU (high CU/high anxiety)) as the independent variable, and psychophysiological or valence/arousal measure during four movie conditions (negative induction, negative suppression, positive induction, and positive suppression) as the repeated measures. Next, univariate ANOVAs were conducted to test the group differences during each movie condition. For psychosocial risk factors, externalizing behavioral measures, and other demographic measures, univariate ANOVAs were conducted with group as the independent variable. Eta-squares were reported as effect sizes. Post hoc comparisons were conducted with Bonferroni corrections. The odds ratio was examined to determine if the groups differed in sex distribution.

## 3. Results

Descriptive statistics for sex, age, IQ, and psychosocial factors are presented in Table 1, along with ANOVA results. The groups did not differ significantly across sex and age, although the high CU/high anxiety group had a significantly lower IQ than the control and anxious groups (*F* (3, 208) = 8.89, *p* < 0.001, *η*^2^ = 0.11).

### 3.1. Emotional Regulation Deficits and CU Variants

**Figure 1:**
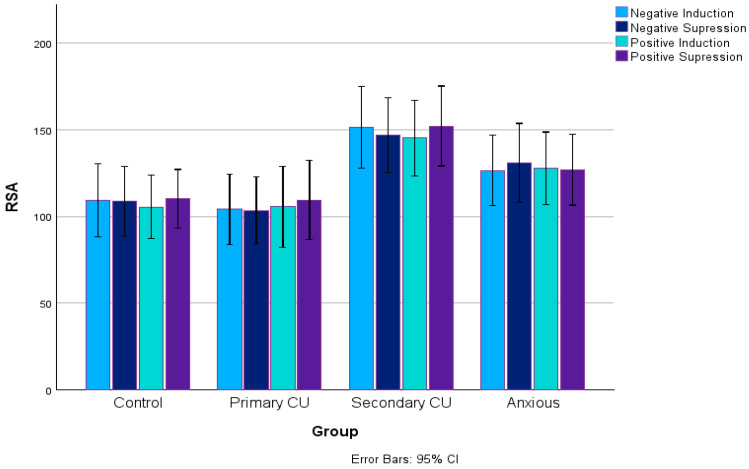
RSA values during the emotional regulation task for the four groups.

For RSA measures, a repeated-measures ANOVA revealed a medium-sized group effect: *F*(3, 116) = 4.09, *p* = 0.008, *η*^2^ = 0.096. Bonferroni-adjusted pairwise comparisons indicated that the high CU/high anxiety group had higher RSA scores than both the control (*p* = 0.019) and high CU/low anxiety groups (*p* = 0.029). There were no significant within-subject effects, *F* < 1, *p* = 0.51, or interaction effects, *F* < 1, *p* = 0.85. Further analyses using univariate ANOVAs for each movie condition also revealed a medium group effect in RSA scores for the negative induction (NI; *F*(3, 125) = 3.28, *p* = 0.023, *η*^2^ = 0.07) and negative suppression (NS; *F*(3, 124) = 2.89, *p* = 0.038, *η*^2^ = 0.07) conditions. All statistics are reported in Table 2. Post hoc analyses using LSD pairwise comparisons indicated that in the NI and NS conditions, the high CU/high anxiety group had greater RSA scores than both the high CU/low anxiety and control groups. Bonferroni-corrected pairwise comparisons were not significant, however. Group effects approached significance for the positive induction (PI; *F*(3, 124) = 2.43, *p* = 0.069, *η*^2^ = 0.06) and positive suppression (PS; *F*(3, 128) = 2.52, *p* = 0.061, *η*^2^ = 0.06) conditions. Post hoc pairwise comparisons with LSD suggested a similar trend that the high CU/high anxiety group had a higher RSA than the high CU/low anxiety and control groups; however, these results did not survive Bonferroni corrections. The RSA values during the emotional regulation task for the four groups are illustrated in Figure 1.

For PEP measures, a repeated-measures ANOVA revealed a small within-subject effect, *F*(1, 147) = 4.28, *p* = 0.04, indicating that participants experienced different PEP reactivities across the movie conditions. There were no significant within-group effects, *F*(2.98, 437.42) = 1.84, *p* = 0.14, *η*^2^ = 0.01, or interaction effects, *F*(8.92, 437.42) = 0.69, *p* = 0.716, *η*^2^ = 0.01. In addition, no significant group effects were observed: *F*(3, 147) = 1.46, *p* = 0.23, *η*^2^ = 0.03. Next, univariate ANOVAs were conducted for each movie condition (NI, NS, PI, and PS), and no significant group effects were observed, *F* < 2, *p* > 0.110.

Repeated-measures ANOVA for self-reported valence did not reveal significant group effects, *F*(3, 204) = 0.41, *p* = 0.75, *η*^2^ = 0.01. Within-subject tests revealed a large effect, *F*(2.39, 486.68) = 417.19, *p* < 0.001, *η*^2^ = 0.67, reflecting differences in valence between the negative emotion film clips and positive emotion film clips, as expected. Univariate ANOVAs for individual movie conditions did reveal a small group effect in the NS condition: *F*(3, 206) = 3.14, *p* = 0.026, *η*^2^ = 0.04. Post hoc LSD pairwise comparisons revealed that the high CU/low anxiety group had higher valence scores than both anxious and high CU/high anxiety groups, suggesting they experienced more positive emotions during the negative suppression (NS) film clip. Bonferroni-adjusted pairwise comparisons showed higher valence scores for the high CU/low anxiety group when compared to the high CU/high anxiety group (*p* = 0.017), but not the anxious group.

Similarly, repeated-measures ANOVA for self-reported arousal levels did not reveal significant group effects, *F*(3, 204) = 0.74, *p* = 0.53, *η*^2^ = 0.01. A small within-subject effect of arousal was observed, *F*(2.45, 498.89) = 5.489, *p* = 0.002, *η*^2^ = 0.03, reflective of a quadratic pattern of arousal across tasks. No further group effects on arousal scores were observed in univariate ANOVAs. Means and standard deviations for the four groups are listed in Table 2.

### 3.2. Psychosocial Risk Factors and CU Variants

The means, standard deviations, and ANOVA results are listed in Table 1. ANOVA revealed that the high CU/high anxiety group had elevated scores on prenatal maternal stress compared to the control and anxious groups (*F*(3, 200) = 5.90, *p* < 0.001, *η*^2^ = 0.08). In addition, compared to the controls, the high CU/high anxiety group had a lower neighborhood collective efficacy (*F*(3, 150) = 4.27, *p* = 0.006, *η*^2^ = 0.08), more physical abuse (F (3, 204) = 5.01, *p* = 0.002, *η*^2^ = 0.12), and verbal abuse (*F*(3, 204) = 10.67, *p* < 0.001, *η*^2^ = 0.25), as well as more domestic violence (verbal: *F*(3, 191) = 10.53, *p* < 0.001, *η*^2^ = 0.14; physical: *F*(3, 191) = 3.55, *p* = 0.016, η^2^ = 0.05). In addition, both the high CU/low anxiety and anxious groups had higher verbal abuse scores than the control group, although they were lower than those of the high CU/high anxiety group. Finally, the high CU/low anxiety group was exposed to more domestic violence (verbal abuse) than the control group.

The groups did not differ significantly on social adversity, *F*(3, 207) = 1.57, *p* = 0.200, *η*^2^ = 0.05. Finally, although the groups did not differ on parent-reported white-collar crime (*F*(3, 199) = 0.27, *p* = 0.84, *η*^2^ = 0.004), the group effect on other crimes approached significance (*F*(3, 200) = 2.61, *p* = 0.05, *η*^2^ = 0.04), with the high CU/high anxiety group scoring higher than the control group.

### 3.3. Externalizing Behaviors

Significant group differences were found for all externalizing behavioral measures, with the high CU/high anxiety group scoring highest among all variables (except for CD symptom count). See Table 1. For aggression and delinquency measures, both CU variants had higher scores than the control group, and the high CU/high anxiety group scored higher than the high CU/low anxiety group (*Fs* > 7.27, *p* < 0.001, *η*^2^ > 0.14). The high CU/high anxiety group scored higher on proactive aggression than the control and anxious groups (*F*(3, 204) = 5.97, *p* < 0.001, *η*^2^ = 0.11). In contrast, for reactive aggression, the high CU/high anxiety and anxious groups scored higher than the control group (*F*(3, 304) = 6.17, *p* < 0.001, *η*^2^ = 0.14). For ADHD symptoms, both CU variants were higher than the control group, and the high CU/high anxiety group also scored higher than the anxious group (*F*(3, 203) = 16.03, *p* < 0.001, *η*^2^ = 0.28). For ODD symptoms, the high CU/high anxiety group scored higher than the control and the anxious group (*F*(3, 203) = 9.84, *p* < 0.001, *η*^2^ = 0.19). For CD symptoms, both variants were higher than the controls and anxious group (*F*(3, 203) = 6.67, *p* < 0.001, *η*^2^ = 0.13). Finally, both variants had higher scores on narcissism and impulsivity than the control and the anxious group (*Fs* > 8.92, *p* < 0.001, *η*^2^ > 0.19).

## 4. Discussion

In this study, we compared the two CU variant groups (high CU/low anxiety and high CU/high anxiety) with two control groups (low CU/low anxiety and low CU/high anxiety) on emotional regulation, along with a variety of psychosocial risk factors and externalizing behavioral measures in a community sample of school-age children. In our sample, the rates of the primary and secondary variants were 13% and 18%, respectively. These rates are comparable to those reported in other studies utilizing community-based samples (see review by [17]). In our study, no significant sex differences were found among the groups, although the high CU/low anxiety group had a slightly higher percentage of males (57%) than the high CU/high anxiety group (41%). This is consistent with prior studies showing a higher proportion of males among the high CU/low anxiety variants and a higher proportion of females among the high CU/high anxiety variants [17]. In addition, we found that the high CU/high anxiety group had a lower IQ than the control and anxious groups, but not the high CU/low anxiety group. Overall, our findings extend our current knowledge of CU variants in several main ways.

First, this was the first study to directly compare emotional regulation capacities between youths with primary or secondary CU profiles and low-risk youth. We hypothesized that emotional dysregulation would characterize the secondary variant (high CU/high anxiety) given its high comorbidity with psychopathologies related to emotional dysregulation. Our findings are broadly consistent with this hypothesis: children with high CU/high anxiety had a higher RSA than those with high CU/low anxiety or low CU/low anxiety, whereas the latter two groups did not differ from each other. In addition, when asked to rate their emotions during movie clips, compared to children with high CU/high anxiety, those with high CU/low anxiety rated the negative movies as more positive, reflecting their deficits in emotional processing. RSA assesses the parasympathetic efference to the heart via the vagus nerve, and atypical RSA levels have been associated with multiple disorders characterized by emotional dysregulation [57], therefore rendering it as a valuable marker of emotional dysregulation vulnerability [58]. In general, moderate RSA suppression is expected to reflect the reduced parasympathetic activity that is necessary for mobilizing resources to complete emotional tasks. In contrast, RSA augmentation indicates increased parasympathetic activity and decreased physiological arousal to allow for a calmer behavioral state [59]. Studies have indicated that antisocial individuals tend to respond with RSA augmentation to emotional stimuli, reflecting their emotional regulation deficits [16]. Such RSA augmentation to emotional stimuli has been linked to ADHD [15], reactive aggression [60,61], and the impulsive–antisocial dimension of psychopathic traits [62]. Links between RSA augmentation and externalizing behavior have also been found. Researchers have observed that RSA augmentation during problem-solving tasks predicted externalizing behaviors in children, particularly in those who also exhibited low basal RSA [63]. Similarly, another study found that RSA augmentation in preschoolers with CU traits in fearful contexts predicted externalizing behavior problems, which was not the case for preschoolers without CU traits [64]. It is therefore hypothesized that RSA augmentation may reflect a loss of control when facing emotional stimuli, which then contributes to impulsive and aggressive tendencies [62].

Alternatively, such RSA augmentation may be a broad indicator of the elevated emotional sensitivity that predisposes children to the negative emotional reactivity seen in children with high CU/high anxiety [3,65]. One prior longitudinal study found that increased RSA reactivity moderated the relationship between childhood adversity and later emotional dysregulation in adolescence [66]. Conradt et al. hypothesized that RSA augmentation was related to a physiological need to remain hypervigilant in unstable or unsafe home environments. The relationship between vagal augmentation, violent or adverse home environments, and externalizing behavior has been reported in several other studies [67,68]. Finally, it is important to acknowledge that RSA augmentation needs to be interpreted in context, and that it is not inherently maladaptive. We are also cautious and note that in our study the RSA differences between the groups were not significant after Bonferroni correction. Nevertheless, given that this is the first study to examine emotional regulation in the two variants, our findings, although preliminary, suggest that emotional dysregulation may uniquely characterize the secondary variants.

Second, previous research consistently demonstrates that individuals with high CU/high anxiety experience greater adversity than their low-anxiety counterparts [22,41]. We support and extend this pattern by showing that children with high CU/high anxiety experienced the highest levels of family adversity, including child physical and verbal abuse, exposure to domestic violence, parent-reported crimes, and low neighborhood collective efficacy, as well as maternal stress during pregnancy. In contrast, we found that children with high CU/low anxiety did not differ from other groups in any psychosocial risk factors, except that they were exposed to more verbal abuse and domestic violence (verbal) than the control group, although they were not as high as the high CU/high anxiety group.

Third, our study showed that children with high CU/high anxiety had the highest scores on all externalizing behavioral measures, including symptoms of ADHD, ODD, and CD, caregiver- and child-reported aggression and delinquency, and levels of narcissism and impulsivity. Although the extant literature highlights greater comorbid psychopathology for the secondary variants than the primary variants ([28,29], although see [21]), we did not find that the two variants differ on psychopathologies or antisocial traits, except for aggression and delinquency. Further, consistent with prior studies [6], the two variants were not differentiated on self-reported levels of proactive and reactive aggression. Finally, we found that children with high CU/high anxiety showed higher levels of psychopathic narcissism than their low-anxiety counterparts. However, this was only significant for youth self-reported symptoms, not caregiver reports. This discrepancy between self- and informant-reports on psychopathic narcissism may reflect insight and perceptual bias in CU populations, with evidence suggesting that self-reported CU may not be as reliable as informant-reports [69]. Further research is needed to determine if psychopathic narcissism may add incremental utility in distinguishing the two variants [70].

The inclusion of the anxious comparison group allowed us to test if children with a high CU/high anxiety experience a double hit of negative outcomes arising from both CU and anxiety. We found some similarities between them and those with low CU traits/high anxiety. Still, compared to the low CU/high anxiety group, the high CU/high anxiety group showed elevated psychosocial adversity (verbal type of child abuse and domestic violence, and prenatal maternal stress) and more externalizing behaviors (ADHD, ODD, CD symptoms, narcissism and impulsivity, aggression, delinquency, and proactive aggression). In fact, they showed additional vulnerabilities compared to youths with either CU or anxiety alone, including more child verbal abuse, elevated narcissism (child report), and more engagement in aggressive and delinquent behaviors, supporting the “double hit” hypothesis [23].

Although an estimated 10–36% of children experience parental maltreatment [20], only a small group develops the complex comorbid clinical profile seen in children with high CU/high anxiety. In our study, we found that the anxious (low CU) group also showed elevated scores on child verbal abuse, child-reported aggression, and reactive aggression. This finding aligns with the proposition that elevated anxiety symptoms may be an alternative response to social adversity in some individuals [3].

The current study has several strengths, which include being the first study to focus on emotional regulation among the CU variants in a community-based mixed-sex sample of children, as well as the inclusion of both caregiver- and self-reports to increase the assessment validity. However, some limitations must be considered when interpreting the results of the study. First, the cross-sectional design of the study prevented the investigation of differences regarding the developmental trajectories of the two variants. Second, it is worth noting that, due to technical issues, psychophysiological data were only available for a subsample. Future studies with larger samples are needed to replicate these findings. Third, it is important to mention that RSA and PEP measures do not directly reflect cognitively mediated regulatory processes, although they offer indirect insight into regulatory capacity. Further, although our emotional regulation paradigm focuses on regulating emotional expression (i.e., hiding or showing emotions on the face), which is a key process of emotional regulation [9], it does not align with the typical conceptualization of emotional regulation that involves intentional regulation strategies such as cognitive reappraisal or distraction. Finally, like most studies in the field, we used a difference score between task and baseline RSA measures to assess emotional regulation capacity. However, such static methods do not capture the temporal dynamics of RSA changes that may contribute unique information [71]. Future psychophysiological studies using dynamic measures and emotional regulation paradigms that include cognitive reappraisal or distraction strategies are needed to replicate and extend our findings.

## 5. Conclusions

Our findings support the theory that the primary CU variant is characterized by low-to-average anxiety levels and no notable history of childhood adversity, whereas the secondary CU variant has pronounced high anxiety levels and a marked history of adverse childhood experiences. These findings extend our knowledge on CU traits by adding preliminary evidence for emotional dysregulation in secondary variants, which may contribute to elevated externalizing behavioral problems including impulsiveness, narcissism, aggression, and delinquency and could be partly attributed to lower IQ. Overall, our findings provide further support to the prior literature indicating that children with high CU/high anxiety exhibit a more severe clinical picture [5,72]. Given the strong associations between prenatal and postnatal environmental risk and secondary variants, it is suggested that early intervention identifying and targeting relevant adverse social conditions may be particularly beneficial for children with high CU/high anxiety [3,73]. Our findings also suggest that these children may benefit greatly from intervention programs that focus on the development of adaptive emotional regulation strategies [12].

## Figures and Tables

**Table 1:** Means and standard deviations for groups on risk exposures, with ANOVA results.

	Low CU		High CU		*F Χ* ^2^	Pairwise Comparisons (w/Bonferroni Correction)
	Low Anxiety (Control; “C”, *n* = 54)	High Anxiety (Anxious; “A”, *n* = 51)	Low Anxiety/Primary (“P”, n = 44)	High Anxiety/Secondary (“S”, *n* = 63)		
Male (%)	42.6	39.2	56.8	41.3	*Χ*^2^= 3.63, *p* = 0.310	-
Age	9.18 (0.68)	8.98 (0.58)	9.05 (0.60)	9.07 (0.62)	*F* (3, 208) = 0.93, *p* = 0.43, *η*^2^ = 0.007	-
IQ	108.26 (19.07)	107.20 (19.10)	98.41 (22.20)	91.92 (18.66)	*F* (3, 208) = 8.89, *p* < 0.001, *η*^2^ = 0.11	A, C > S
Prenatal and Postnatal Adversity						
Prenatal Maternal Stress	0.37 (0.73)	0.60 (0.78)	0.79 (0.97)	1.14 (1.34)	*F* (3, 200) = 5.90, *p* < 0.001. *η*^2^ = 0.08	S > A, C
Social Adversity	2.61 (1.88)	3.33 (2.22)	3.23 (1.94)	3.32 (2.05)	*F* (3, 207) = 1.57, *p* = 0.200, *η*^2^ = 0.05	-
Parent White-Collar Crime	8.50 (5.83)	8.90 (5.11)	8.82 (6.31)	7.94 (6.77)	*F* (3, 199) = 0.27, *p* = 0.840, *η*^2^ = 0.004	-
Other Parental Crime	4.05 (4.65)	5.67 (5.07)	4.87 (6.26)	7.13 (7.59)	*F* (3, 200) = 2.61, *p* = 0.050, *η*^2^ = 0.04	S > C
Neighborhood Collective Efficacy	34.15 (6.96)	29.74 (9.14)	30.55 (8.91)	27.50 (9.17)	*F* (3, 150) = 4.37, *p* = 0.006, *η*^2^ = 0.08	C > S
Domestic Violence (Physical)	0.43 (1.87)	1.21 (3.31)	1.88 (5.50)	3.81 (8.78)	*F* (3, 191) = 3.55, *p* = 0.016, *η*^2^ = 0.05	S > C
Domestic Violence (Verbal)	4.57 (5.65)	5.81 (5.35)	8.05 (6.73)	10.90 (6.94)	*F* (3, 191) = 10.53, *p* < 0.001, *η*^2^ = 0.14	S > A, C; P > C
Childhood Abuse (Physical)	1.57 (2.47)	2.45 (2.60)	3.35 (3.36)	4.17 (5.40)	*F* (3, 204) = 5.01, *p* = 0.002, *η*^2^ = 0.12	S > C
Childhood Abuse (Verbal)	2.69 (3.06)	5.02 (4.60)	4.88 (4.48)	7.33 (5.09)	*F* (3, 204) = 10.67, *p* < 0.001, *η*^2^ = 0.25	S > A, P > C
Externalizing Behaviors						
Aggression (Parent Report)	1.33 (1.88)	3.59 (3.75)	5.84 (4.96)	8.56 (6.06)	*F* (3, 208) = 27.14, *p* < 0.001, *η*^2^ = 0.34	S > P > C; S > A
Aggression (Child Report)	5.00 (3.33)	8.63 (4.31)	7.11 (3.53)	10.08 (5.03)	*F* (3, 208) = 15.45, *p* < 0.001, *η*^2^ = 0.31	S, A >C; S > P
Delinquency (Parent Report)	0.69 (1.08)	1.20 (1.31)	2.50 (1.85)	3.86 (3.66)	*F* (3, 208) = 21.62, *p* < 0.001, *η*^2^ = 0.37	S > P > A, C
Delinquency (Child Report)	2.65 (1.42)	3.31 (2.01)	2.82 (1.87)	4.29 (2.66)	*F* (3, 208) = 7.27, *p* < 0.001, *η*^2^ = 0.14	S > C, P
Proactive Aggression	0.53 (1.05)	1.08 (2.31)	1.94 (3.56)	2.63 (3.62)	*F* (3, 204) = 5.97, *p* < 0.001, *η*^2^ = 0.11	S > A, C
Reactive Aggression	4.05 (3.35)	6.38 (3.40)	6.18 (4.82)	7.22 (4.47)	*F* (3, 304) = 6.17, *p* < 0.001, *η*^2^ = 0.14	S, A > C
ADHD Symptoms	2.80 (3.06)	5.04 (3.82)	6.95 (4.64)	8.52 (6.07)	*F* (3, 203) = 16.03, *p* < 0.001, *η*^2^ = 0.28	S, P > C; S > A
ODD Symptoms	2.59 (2.54)	2.69 (2.57)	3.93 (2.98)	5.18 (3.43)	*F* (3, 203) = 9.84, *p* < 0.001, *η*^2^ = 0.19	S > A, C
CD Symptoms	0.30 (0.69)	0.43 (0.96)	1.17 (1.71)	1.13 (1.52)	*F* (3, 203) = 6.67, *p* < 0.001, *η*^2^ = 0.13	S, P > A, C
Narcissism (Parent Report)	0.69 (0.85)	1.28 (1.11)	2.30 (1.97)	3.10 (2.51)	*F* (3, 203) = 19.92, *p* < 0.001, *η*^2^ = 0.34	S, P > A, C
Narcissism (Child Report)	1.20 (1.47)	2.08 (1.76)	2.30 (2.08)	3.62 (2.94)	*F* (3, 201) = 11.78, *p* < 0.001, *η*^2^ = 0.20	S > P, A, C
Impulsivity (Parent Report)	1.48 (1.13)	1.90 (1.36)	2.89 (1.81)	3.64 (1.89)	*F* (3, 203) = 20.93, *p* < 0.001, *η*^2^ = 0.26	S, P > A, C
Impulsivity (Child Report)	1.49 (1.45)	2.34 (1.44)	2.52 (1.72)	3.12 (1.94)	*F* (3, 201) = 8.92, *p* < 0.001, *η*^2^ = 0.19	S, P > C

Note: A = anxious group; C = control group; P = primary variants; S = secondary variants.

**Table 2:** Means and standard deviations for groups on emotional regulation measures, with ANOVA results.

	Low CU		High CU		F	Pairwise Comparisons
	Low Anxiety (Control; “C”, *n* = 34–46)	High Anxiety (Anxious; “A”; *n* = 35–46)	Low Anxiety/Primary (“P”; *n* = 22–36)	High Anxiety/Secondary (“S”; *n* = 38–53)		
RSA (Negative Induction)	109.15 (58.27)	121.64 (55.05)	104.17 (45.84)	145.51 (67.31)	*F* (3, 125) = 3.28, *p* = 0.023, *η*^2^ = 0.07	LSD: S > C, P
RSA (Negative Suppression)	108.87 (55.93)	127.13 (60.17)	108.87 (49.64)	144.79 (61.54)	*F* (3, 124) = 2.89, *p* = 0.038, *η*^2^ = 0.07	LSD: S > C, P
RSA (Positive Induction)	109.82 (56.71)	126.79 (56.77)	110.85 (57.26)	143.61 (62.41)	*F* (3, 124) = 2.43, *p* = 0.069, *η*^2^ = 0.06	-
RSA (Positive Suppression)	113.13 (50.72)	123.12 (53.31)	113.96 (54.81)	146.25 (66.57)	*F* (3, 128) = 2.52, *p* = 0.061, *η*^2^ = 0.06	-
PEP (Negative Induction)	77.96 (19.24)	88.41 (19.76)	80.50 (22.72)	83.33 (22.7)	*F* (3, 175) = 2.04, *p* = 0.110, *η*^2^ = 0.03	-
PEP (Negative Suppression)	81.44 (20.12)	88.57 (18.84)	83.75 (15.7)	85.21 (24.21)	*F* (3, 175) = 0.89, *p* = 0.450, *η*^2^ = 0.02	-
PEP (Positive Induction)	78.38 (19.12)	90.32 (20.77)	84.15 (21.79)	88.08 (21.79)	*F* (3, 175) = 2.41, *p* = 0.069, *η*^2^ = 0.04	-
PEP (Positive Suppression)	81.55 (19.00)	89.16 (19.66)	83.21 (24.13)	87.62 (23.44)	*F* (3, 171) = 1.21, *p* = 0.309, *η*^2^ = 0.07	-
Valence (Negative Induction)	2.15 (0.97)	2.10 (0.97)	2.02 (1.05)	2.02 (1.09)	*F* (3, 206) = 0.22, *p* = 0.870, *η*^2^ = 0.003	-
Valence (Negative Suppression)	2.25 (1.07)	2.16 (1.06)	2.64 (1.14)	1.98 (1.11)	*F* (3, 206) = 3.14, *p* = 0.026, *η*^2^ = 0.04	LSD: S, A < P Bonferroni: S < P
Valence (Positive Induction)	4.34 (0.90)	4.43 (0.82)	4.23 (0.91)	4.44 (0.93)	*F* (3, 205) = 0.61, *p* = 0.608, *η*^2^ = 0.01	-
Valence (Positive Suppression)	4.65 (0.56)	4.65 (0.86)	4.43 (0.85)	4.56 (0.98)	*F* (3, 204) = 0.75, *p* = 0.526, *η*^2^ = 0.01	-
Arousal (Negative Induction)	1.94 (1.06)	2.16 (1.85)	2.50 (1.40)	2.21 (1.44)	*F* (3, 206) = 1.18, *p* = 0.318, *η*^2^ = 0.02	-
Arousal (Negative Suppression)	2.47 (1.31)	2.68 (1.22)	2.86 (1.34)	2.49 (1.39)	*F* (3, 206) = 0.95, *p* = 0.418, *η*^2^ = 0.01	-
Arousal (Positive Induction)	2.34 (1.41)	2.14 (1.37)	2.20 (1.42)	2.41 (1.59)	*F* (3, 205) = 0.38, *p* = 0.765 *η*^2^ = 0.01	-
Arousal (Positive Suppression)	2.17 (1.32)	2.12 (1.35)	2.43 (1.56)	2.11 (1.35)	*F* (3, 204) = 0.55, *p* = 0.648 *η*^2^ = 0.01	-

Note: A = anxious group; C = control group; P = primary variants; S = secondary variants. RSA = respiratory sinus arrhythmia; PEP = pre-ejection period.

## Data Availability

Data will be made available upon reasonable request. The data are not publicly available due to specific ethical and privacy considerations.
